# Expression Profiling of Autophagy Genes *BxATG1* and *BxATG8* under Biotic and Abiotic Stresses in Pine Wood Nematode *Bursaphelenchus xylophilus*

**DOI:** 10.3390/ijms18122639

**Published:** 2017-12-06

**Authors:** Fan Wu, Li-Na Deng, Xiao-Qin Wu, Hong-Bin Liu, Jian-Ren Ye

**Affiliations:** 1 Co-Innovation Center for Sustainable Forestry in Southern China, College of Forestry, Nanjing Forestry University, Nanjing 210037, China; huihui5541@sina.com (F.W.); denglina121@163.com (L.-N.D.); Hongbin219@gmail.com (H.-B.L.); jrye@njfu.edu.cn (J.-R.Y.); 2 Jiangsu Key Laboratory for Prevention and Management of Invasive Species, Nanjing Forestry University, Nanjing 210037, China; 3 College of Marine and Biological Engineering, Yancheng Institute of Technology, Yancheng 224003, China

**Keywords:** *Bursaphelenchus xylophilus*, autophagy, autophagy-related genes, quantitative reverse transcription PCR, RNA interference

## Abstract

The pine wood nematode (PWN), *Bursaphelenchus* *xylophilus*, is the pathogen of pine wilt disease (PWD) and causes huge economic losses in pine forests and shows a remarkable ability to survive under unfavorable and changing environmental conditions. This ability may be related to autophagy, which is still poorly understood in *B.* *xylophilus*. Our previous studies showed that autophagy exists in PWN. Therefore, we tested the effects of autophagy inducer rapamycin on PWN and the results revealed that the feeding rate and reproduction were significantly promoted on fungal mats. The gene expression patterns of *BxATG1* and *BxATG8* under the different stress were determined by quantitative reverse transcription PCR (qRT-PCR). We tested the effects of RNA interference on *BxATG1* and *BxATG8* in PWN during different periods of infection in *Pinus thunbergii*. The results revealed that *BxATG1* and *BxATG8* may play roles in allowing PWN to adapt to changing environmental conditions and the virulence of PWN was influenced by the silence of autophagy-related genes *BxATG1* and *BxATG8*. These results provided fundamental information on the relationship between autophagy and PWN, and on better understanding of gene function of *BxATG1* and *BxATG8* in PWN.

## 1. Introduction

Pine wilt disease (PWD) was considered as “pine cancer”, which is caused by pine wood nematode (PWN), *Bursaphelenchus xylophilus*. The first case of PWD caused by *B. xylophilus* in Europe was reported in 1999 in Portugal [1], and was soon after found in Spain [2]. The PWD was introduced to Japan a century ago and spread to China and Korea in the 1980s [3]. *B. xylophilus* usually damages exotic pine trees and causes serious damage and great economic loss to the resources of pine trees and natural landscapes in most areas of China [4]. At present, research on PWN mainly focuses on the pathogenic mechanism, inspection, quarantine and how to identify PWN and other species of *Bursaphelenchus* [5]. A number of studies have focused on the pathogenic and growth related genes in PWN [6]. However, the pathogenic mechanism of PWN is not yet clear. As an invasive species, how PWN can fit some environmental conditions, such as high and low temperatures, and response to the resistance reaction of pine tree becomes a key reason for the death of pine trees. These are still open questions in PWD research.

Autophagy is a self-protection for an eukaryotic cell to adapt poor conditions. Several studies have shown that autophagy plays significant roles in physiological and pathophysiological processes in eukaryotic cells [7,8]. Autophagy related genes are indispensable for the ability to drop in each growing phase of *Pyricularia grisea*, such as production of spores, turgor of appressorium, pathogenicity and sexual reproduction [9,10,11,12]. Interference of some autophagy related genes will produce some growth defects. For example, the shape of dauer larvae will be affected; the life-span will be shortened; survival rate at starvation will be reduced; and the cell will be boosted to die [13]. In recent years, some functional genes similar to autophagy genes have been identified in *Caenorhabditis elegans* [14,15,16]. Atg1 is a protein kinase, which can activate the key inducing factor for autophagy [17]. However, the autophagy gene *Atg8* participates in two ubiquitin similar integration systems and it is the most direct proof to be always used to test the autophagic activity [18]. Target of rapamycin (TOR) is an evolutionarily conserved serine/threonine protein kinase that was first found in yeast and subsequently its homologs were found in mammals. They were collectively referred to as mammalian target of rapamycin (mTOR). The mTOR signaling pathway plays an important role in the regulation of cell growth, proliferation, apoptosis and autophagy. Under normal conditions, the mTOR pathway is activated and autophagy is inhibited. When this pathway is repressed, autophagy is activated [19]. Rapamycin (RAPA) is the first known mTOR pathway inhibitor. The study found that rapamycin (RAPA) was a potential autophagy inducer that could induce and promote autophagy by inhibiting the mTOR pathway [20]. However, there are no previous reports on mTOR in PWN. Induced autophagy and impact on the PWN remain poorly understood. In natural conditions, autophagy occurs at a low level. When the external environment changes, the level of autophagy will change to regulate the homeostasis of the organism [8]. This process is likely to have a certain impact on the environmental adaptability and pathogenicity of PWN. In recent research in our laboratory, the phenomenon of autophagy was found to exist in PWN and two autophagy genes *BxATG1* and *BxATG8* of PWN were cloned and analyzed biologically [21]. However, reports on autophagy of PWN are scarce and the relationship between autophagy genes and the pathogenicity of PWN is still unclear. Based on previous research, an exogenous autophagy inducer rapamycin was introduced in this study to explore the relationship between feeding, reproduction and autophagy in PWN. Quantitative reverse transcription PCR (qRT-PCR) was used to quantify the expression of autophagy genes *BxATG1* and *BxATG8* of PWN when temperature changes and oxidation occurs. The aim of the study was to reveal the relationship between autophagy and anti-reversion force of PWN, and to provide a reference to elucidate the pathogenesis of PWN.

## 2. Results

### 2.1. Effect of Rapamycin on B. xylophilus Feeding Rate and Reproduction on Fungal Mats

**Figure 1:**
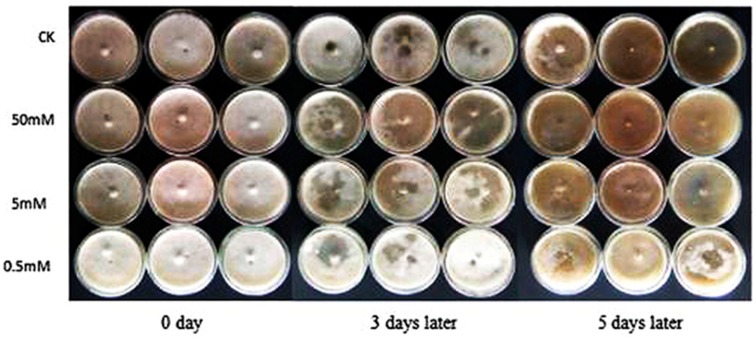
Effect of rapamycin on the pine wood nematode (PWN) inoculated onto *B. cinerea*. The nematodes treated by ddH_2_O control solution (CK) and 50, 5, and 0.5 mM rapamycin were grown in *B. cinerea*.

The effect of rapamycin on PWN reproduction was tested on Potato Dextrose Agar (PDA) plates inoculated with *Botrytis cinerea* at 25 °C. After the nematode was treated by rapamycin, the feeding rate of nematode was faster than control. The feeding rate increased with an increase of rapamycin concentration and the treatment of 50 mM was the fastest. These results showed that the feeding rate of PWN was significantly influenced by rapamycin treatment, and PWN increases its feeding rate at the autophagy induction at 50 mM concentration of rapamycin solution (Figure 1).

**Figure 2:**
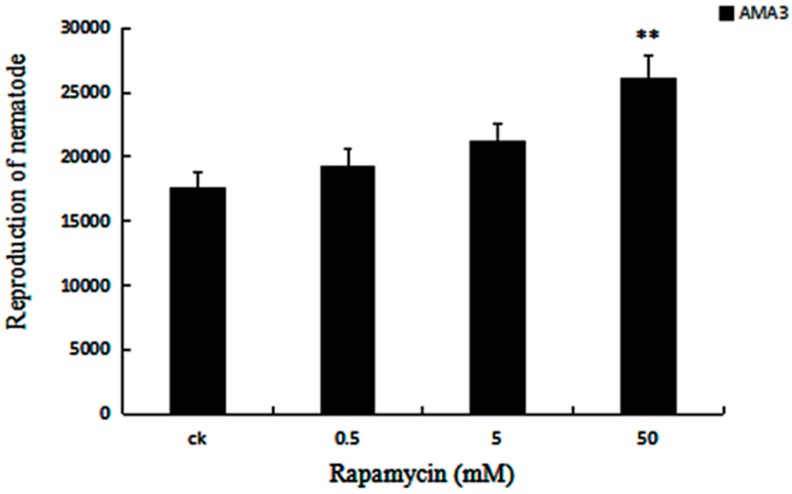
Effect of Rapamycin on reproduction of PWN. Reproduction rate of PWN washed from PDA plate of *B. cinerea* (ddH_2_O and 0.5, 5, and 50 mM Rapamycin). Data represent mean ± SD from three independent experiments. Bars show standard deviations of the mean. Asterisks on top of the bars indicating statistically significant differences between the Rapamycin-treated (0.5, 5 and 50 mM Rapamycin) nematodes and controls (** *p* < 0.01, Student’s *t*-test).

After treated with rapamycin solution, the PWN grew more rapidly than that of the control, and the one, which was treated with 50 mM rapamycin solution was the highest, and other treatments were slightly higher, and the amplitude of fluctuation was about 5000 pieces of nematodes (Figure 2). The result manifested autophagy plays a boost role in the reproduction of PWN. This was the same trend of feeding rate.

### 2.2. Expression Level of Autophagy Genes BxATG1 and BxATG8 in B. xylophilus after Treated with Rapamycin

**Figure 3:**
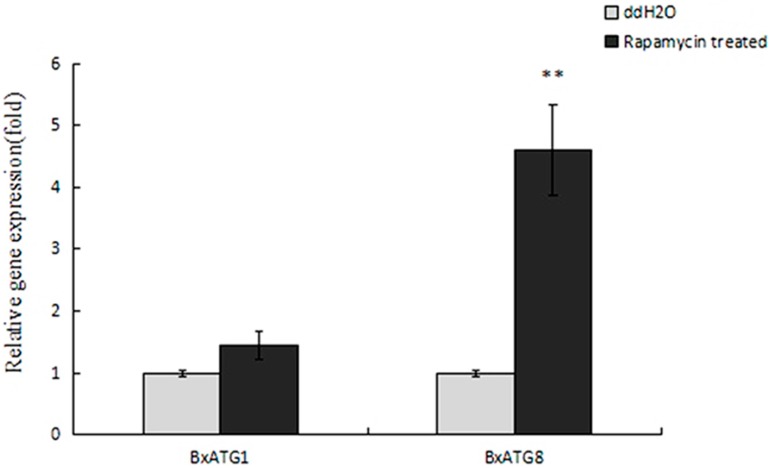
The expression level of *BxATG1* and *BxATG8* after treatment with a 50 mM concentration of rapamycin. Data represent mean ± SD from three independent experiments. Bars show standard deviations of the mean. Asterisks on top of the bars indicate statistically significant differences (** *p* < 0.01, Student’s *t*-test).

Rapamycin was used to induce Autophagy. Quantitative reverse transcription PCR (qRT-PCR) was performed to determine the effect of rapamycinon the expression of *BxATG1* and *BxATG8* expression levels. The gene expression of *BxATG1* and *BxATG8* significantly increased after PWN was soaking in a 50 mM concentration of rapamycin solution compared with those after PWN was soaking in ddH_2_O control solution. When the expression level of the control was considered as 100%, the mean expression level of rapamycin-treated samples rose by 1.45 times and 4.60 times (Figure 3). The autophagy-related gene *Atg8* was the marker of autophagy. Thus, these results showed that rapamycin can induce autophagy of PWN effectively.

### 2.3. Response and Expression of B. xylophilus Autophagy Genes BxATG1 and BxATG8 at Temperature Changes

**Figure 4:**
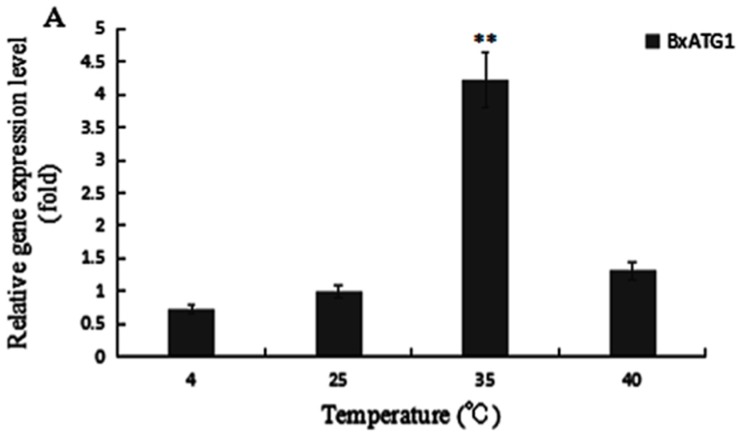

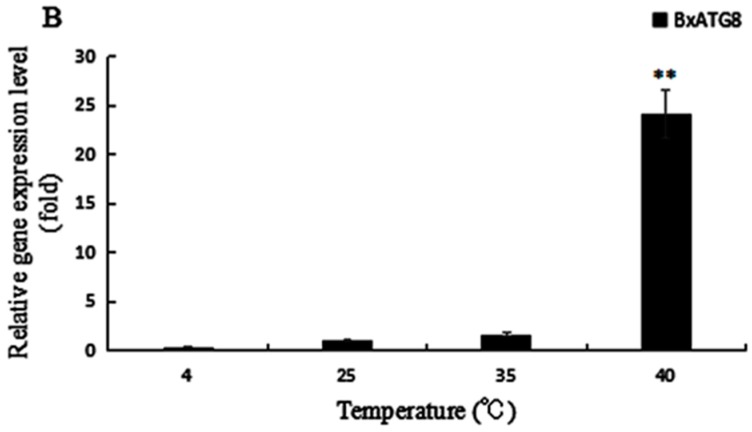
Quantitative reverse transcription PCR (qRT-PCR) analysis of: *BxATG1* (**A**); and *BxATG8* (**B**), in *PWN* after treated at different temperatures. The expression level of 25 °C was set as 100%. Data represent mean ± SD from three independent experiments. Bars show standard deviations of the mean. Asterisks on top of the bars indicate statistically significant differences (** *p* < 0.01, Student’s *t*-test).

The expression level of gene *BxATG1* of PWN AMA3 increased with increasing temperature and reached the peak when the temperature was at 35 °C. It showed that the autophagy reaction was most fiercely enabled when the nematode was at 35 °C. It increased by 4.2 times of the expression level when at 25 °C. When the temperature was 40 °C, the expression level of gene *BxATG1* declined suddenly (Figure 4A).

The expression level of gene *BxATG8* of AMA3 increased in a trapezoid with increasing temperature. The expression level of gene *BxATG8* reached the highest at 40 °C and rose by 24.1 times of the one at 25 °C. *ATG8* is the main gene to be measured for autophagy activity (Figure 4B). When the two genes were compared with each other, the expression level of gene *BxATG8* was much higher than that of gene *BxATG1*. When both were at peaks, the peak expression level of gene *BxATG8* of nematode AMA3 was 24 times of the one at the most suitable growing temperature, but the gene *BxATG1* was only four times. The results showed that PWN goes through the high temperature stress by regulating autophagy activity and *ATG8* plays an important role in the process of regulating autophagy.

### 2.4. Expression Level of B. xylophilus Autophagy Genes BxATG1 and BxATG8 under Oxidative Stress

**Figure 5:**
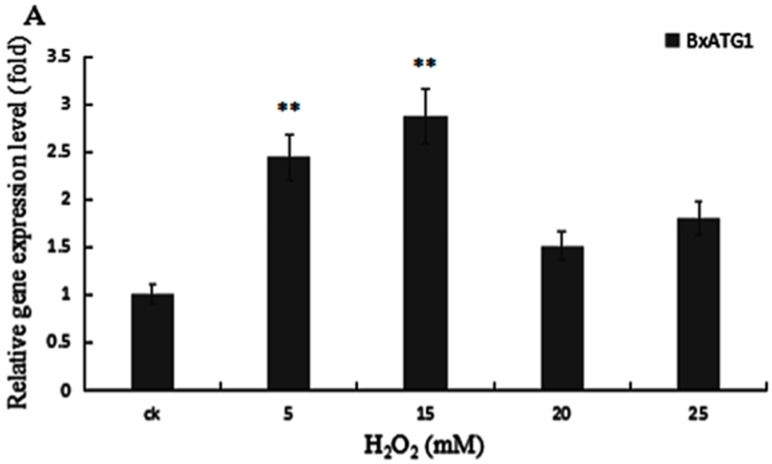

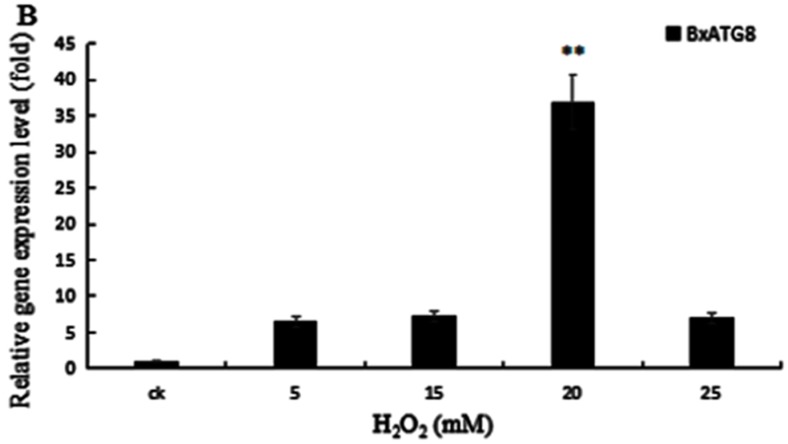
Quantitative reverse transcription PCR (qRT-PCR) analysis of: *BxATG1* (**A**); and *BxATG8* (**B**), in PWN after treated with oxidative stress. The expression level of the control was set as 100%. Data represent mean ± SD from three independent experiments. Bars show standard deviations of the mean. Asterisks on top of the bars indicate statistically significant differences (** *p* < 0.01, Student’s *t*-test).

Compared to CK (0 mM H_2_O_2_), with an increase of H_2_O_2_ concentration, the expression level of autophagy related gene *BxATG1* of PWN increased first and then declined. When the concentration reached 15 mM, the starting reaction of autophagy was the strongest. The expression level of *BxATG1* increased by 2.87 times, compared with the counterpart, then it descended (Figure 5A).

The expression level of autophagy related gene *BxATG8* of PWN ascended with the increase of H_2_O_2_ concentration and then decreased. When the concentration reached 20 mM, the autophagy response was strongest. The expression level of *BxATG8* increased 36.9 times compared to the check (Figure 5B). The results showed that PWN helped themselves through the oxidative stress by regulating autophagy activity.

### 2.5. Expression Level of B. xylophilus Autophagy Genes BxATG1 and BxATG8 after Pine Trees Inoculated with B. xylophilus

**Figure 6:**
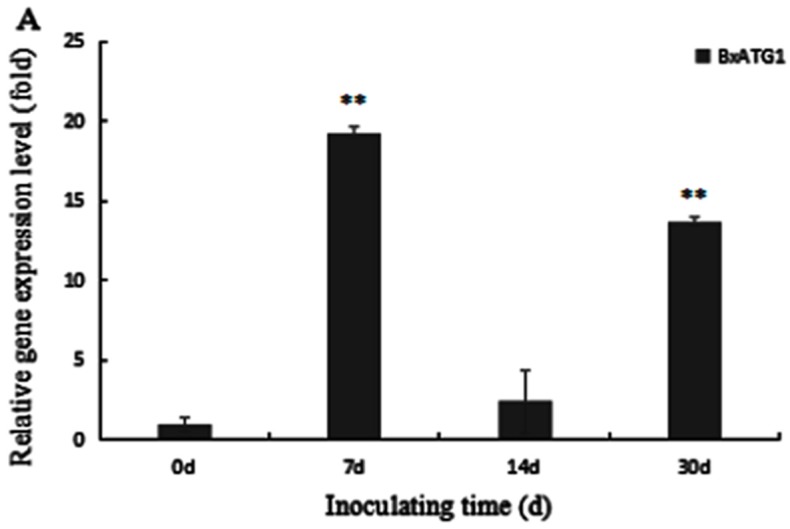

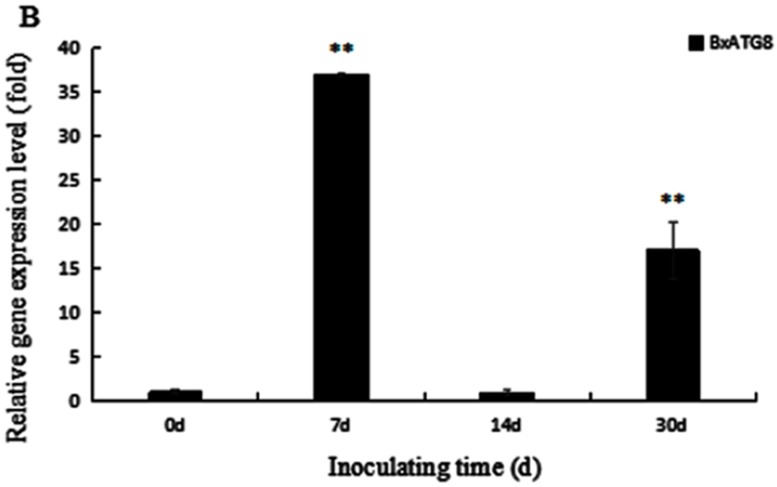
Quantitative reverse transcription PCR (qRT-PCR) analysis of: *BxATG1* (**A**); and *BxATG8* (**B**), in PWN after inoculating trees. The expression level of the control was set as 100%. Data represent mean ± SD from three independent experiments. Bars show standard deviations of the mean. Asterisks on top of the bars indicate statistically significant differences (** *p* < 0.01, Student’s *t*-test).

Two-year-old *Pinus thunbergii* seedlings were inoculated with PWN. Expression of autophagy genes *BxATG1* and *BxATG8* in PWN reached the highest level after the nematode invading pine trees for seven days. Gene *BxATG1* increased by 19.3 times and gene *BxATG8* increased by 36.9 times. After 14 days, the expression of genes *BxATG1* and *BxATG8* were only 2.4 times higher for gene *BxATG1* and 0.89 times lower for gene *BxATG8*. After pine trees were inoculated for 30 days, expression of *BxATG1* and *BxATG8* presented second peaks, which were 13.7 and 17.0 times, respectively. Meanwhile, the expression of *BxATG8* at peak was higher than that of gene *BxATG1* (Figure 6). The result showed autophagy was strong in the beginning and the last period of invading pine tree.

### 2.6. Virulence of Pine Wood Nematode (PWN) after RNAi

**Figure 7:**
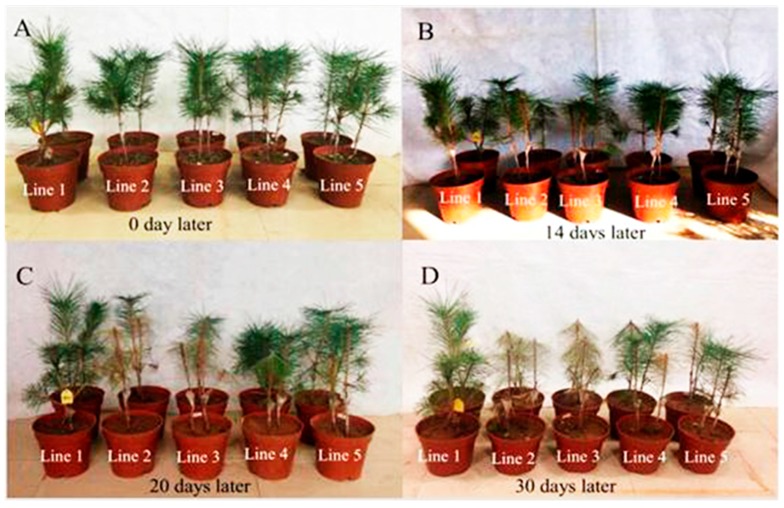
Symptoms in *P. thunbergii* seedlings: 0 day (**A**); 14 days (**B**); 20 days (**C**); and 30 days (**D**) after inoculation with nematodes soaked in ddH_2_O (line 2), ds*GFP* (line 3), d*sBxATG1* (line 4), and ds*BxATG8* (line 5) solutions. *P. thunbergii* seedlings treated with ddH_2_O alone were considered as controls (line 1). Each pot contains two *P. thunbergii* seedlings.

Two-year-old *Pinus thunbergii* seedlings were inoculated with dsRNA- and ddH_2_O-treated nematodes (Figure 7A). After 14 days, wilting appeared in the seedlings inoculated with nematodes soaked in the negative controls (ds*GFP*-treated and ddH_2_O-treated solution) (Figure 7B), and the infection rates were both 50%. The disease severity indices (DSI) were both 12.5. After 20 days, the seedlings inoculated with nematodes soaked in ds*BxATG1* and ds*BxATG8* solutions began to wilt (Figure 7C); the infection rates of *P. thunbergii* inoculated with nematodes soaked in ds*BxATG1*, ds*BxATG8* and ds*GFP* and ddH_2_O were 50%, 75%, 100% and 100%, respectively, and the DSI were 12.5, 18.8, 50 and 50, respectively. After 30 days, all of the seedlings inoculated with ds*GFP*-treated and ddH_2_O-treated nematodes turned red-brown (Figure 7D), and DSI were both 100, and the disease severity indices of *P. thunbergii* seedlings inoculated with nematodes soaked in ds*BxATG1* and ds*BxATG8* were 56.3 and 87.5, respectively (Table 1). The wilting processes of *P. thunbergii* seedlings were different among the treatments. *P. thunbergii* seedlings inoculation with ds*GFP*-treated and ddH_2_O-treated nematodes started to wilt in 12 days, and were dead in 29 and 28 days, respectively. Delayed symptom development was observed by silencing *BxATG1* and *BxATG8*, respectively (Table 1). The results indicated that the virulence of PWN was influenced by the RNAi of autophagy genes *BxATG1* and *BxATG8*.

## 3. Discussion

Recently, the function of autophagy in eukaryote is explored and indicated that it played an important biological role. Reports showed that autophagy can boost reproduction of *Aedes aegypti*, *Pyricularia grisea* and *Colletotrichum orbiculare*, and play a role in promoting adaptive ability in stress environment [22,23]. In this study, the feeding rate of PWN increased in trapezoid with the ascending of induction rapamycin concentration and the trend of reproduction is very similar with the feeding rate. The result confirmed that autophagy plays a role in promoting the feeding and reproductive abilities of PWN*.*

Autophagy is helpful for organism to live in an adverse environment and improve its adaptability [24]. As a defensive reaction, the process of autophagy will degrade the damaged protein, organelle and cytoplasmic components under environmental stress, provide nutrition, keep living for cells, and provide raw materials for self-repair and existing cells [25]. Autophagy occurs due to the stimulation of ambient environment such as temperature changes [13]. Active oxygen is also one of the induction factors of autophagy [26,27]. At the beginning of PWN invading pine tree, active oxygen increased sharply in the pine trees [28,29,30]. *Atg* genes can perform different functions [31,32]. Quantitative reverse transcription PCR (qRT-PCR) was performed to analyze the effect of temperature change and oxidative stress on the *BxATG1* and *BxATG8* expression levels. Based on our results, with the increase of temperature, the expression level of autophagy genes *BxATG1* and *BxATG8* of PWN ascended and reached peak, and high temperature stimulated autophagy of PWN. The results also suggested PWN adapted to the environment of oxidative stress through autophagy and the expression level of genes *BxATG1* and *BxATG8* reached the peak with the ascending of H_2_O_2_ concentration. PWN could die later for the possibilities of too much autophagy and high concentration of active oxygen. It is not difficult to find that, whether the nematode was under stress of high temperature or oxidation, the peak of expression level of gene *BxATG8* always occurred after gene *BxATG1*. This conforms to the sequence found in eukaryotes (e.g., yeast) and *C. elegans* that gene *Atg1* participated in the induction of autophagy and gene *Atg8* participated in ubiquitin similar integrative system [33]. Of course, this is only speculated from the sequence of gene expressions. Whether upstream and downstream signal channels, similar to that of *C. elegans*, exist in PWN, and the complex signal transmission and molecular regulation mechanism in autophagy of PWN [34] should be further studied.

To resist the invasion by pine wood nematodes, the pine induces defense mechanisms against the pathogen and numerous defense molecules are generated, such as cyclic aromatics, terpenoids and reactive oxygen species [35,36,37]. At the same time, PWN must also mobilize reciprocal defensive reactions to avoid damage from the complex compounds [38]. Autophagy is part of the cellular response, which can change rapidly in a cell due to changing environmental and physiological conditions. Therefore, it plays an important role in the cellular response to stress [39]. Autophagy is a means of clearance that reduces damage caused by the plant’s molecular responses [40,24]. In the present study, we found that autophagy in PWN reacted strongly at the beginning and at the end of infecting *P. thunbergii*. We presumed that pine wood nematode adapted a variety of resistance of pine by strong autophagy in the early stages of infection. The autophagy reaction reached a second peak because of lack of food and high population density in the late stage of infection. RNAi was developed as an effective tool in plants and animals to study gene functions and for genetic manipulation [41,42]. Moreover, RNAi has also been used to assess the pathogenic and molecular effects of silenced *B. xylophilus* genes [43,44,45,46]. In the present study, *BxATG1* and *BxATG8* were shown to likely be associated with virulence in *B. xylophilus* by eliminating damage caused by *P. thunbergii* defense mechanisms and promoting the synthesis of toxic metabolites to help *B. xylophilus* colonize the pine trees. However, the potential role of autophagy in PWN needs to be further investigated.

In summary, autophagy can improve the feeding and breeding abilities of PWN. We found from the molecular level that anti-adverse force and pathogenicity of PWN are related with the regulation mechanism of autophagy genes. It is speculated that, by autophagy, PWN can regulate its internal environment to survive adverse conditions such as high temperatures or oxidative stress, minimize the damage resulted from defensive mechanism of pine and promote synthesis of its toxin metabolite, to help successfully invade and establish colonies that damage pine trees. The discovery of these relationships between autophagy and PWN will assist us to understand the biological suitability mechanism of PWN under adverse conditions and the function of autophagy genes in pine wilt disease.

## 4. Materials and Methods

### 4.1. PWN Growth Conditions and Experimental Organisms

The highly virulent AMA3 strain of *PWN* was isolated from wood chips of infested *P. thunbergii* Parl from Anhui, China. The nematodes were grown in colonies of *B. cinerea* Pers [47] on PDA plates for 7 days at 25 °C. Then, they were extracted overnight from PDA plates using the Baermann funnel method [48]. Two-year-old *P. thunbergii* seedlings were obtained from the greenhouse at Nanjing Forestry University (Nanjing, China). 

### 4.2. Preparation of Autophagy Inducer

Fifty milligrams of rapamycin were poured into 1 mL DMSO to be 50 mg/mL (about 50 mM) solution, which was then diluted with ddH_2_O (Double-distilled H_2_O) into 5 mM and 0.5 mM for use [49,50].

### 4.3. Analysis of Feeding and Reproduction of PWN after Autophagy Induction

One thousand nematodes from different treatments (ddH_2_O and 50, 5, 0.5 mM rapamycin) were picked and transferred onto a PDA plate with *B. cinerea* and cultured at 25 °C for 5 days. Three biological replicates were used in each treatment. The growth condition of nematodes was photographed daily. Subsequently, the nematodes were extracted from PDA plates using the Baermann funnel method and the nematodes were counted.

### 4.4. RNA Extraction and cDNA Synthesis of PWN

The total RNA of collected nematodes (a mixture of adults and juveniles) was extracted using TRIzol reagent (Invitrogen, Waltham, MA, USA). The RNA was quantified using a spectrophotometer and examined by electrophoresis on a 1% agarose gel. cDNA was synthesized from 2 µg of total RNA using the TransScript II One-Step gDNA Removal and cDNA Synthesis SuperMix according to the manufacturer’s instructions (Trans Gen Biotech, Beijing, China).

### 4.5. Quantitative Reverse Transcription PCR (qRT-PCR)

Quantitative reverse transcription PCR (qRT-PCR) was performed to determine the expression levels of *BxATG1* and *BxATG8*. qRT-PCR was then carried out using SYBR Green Master Mix (Vazyme, Nanjing, China). The Actin gene of PWN was used as an internal control, with the primers listed in Table 2. Relative expression levels were determined using the ABI Prism 7500 software (Applied Biosystems, Foster City, CA, USA) and the 2^−ΔΔ*C*t^ method. qRT-PCR was conducted with three biological and technical replicates [51].

### 4.6. Analysis of Expression Level of BxATG1 and BxATG8 When Temperature Changes

Collected nematodes with different treatment (4, 25, 35 and 40 °C water bath for 15 min) were transferred into Eppendorf (EP) pipes. Then RNA of PWN was extracted and cDNA was synthesized (see Section 4.4)*.* qRT-PCR was performed to determine the expression levels of *BxATG1* and *BxATG8*. 

### 4.7. Analysis of Expression Level of BxATG1 and BxATG8 in Oxidative Stress

Nematodes were soaked in different H_2_O_2_ solutions (1 mL ddH_2_O with 10 µL volumes of 5, 15, 20 and 25 mM H_2_O_2_) for 30 min. RNA was extracted and synthesize cDNA of *PWN* was synthesized (see Section 4.4). qRT-PCR was performed to determine the expression levels of *BxATG1* and *BxATG8.*

### 4.8. Analysis of Expression Level of BxATG1 and BxATG8 after Pine Was Inoculated with B. xylophilus

The nematodes were inoculated to *P. thunbergii* seedlings. One thousand nematodes were injected into each seedling [52]. Nematodes were extracted overnight from *P. thunbergii* seedlings (after inoculating 7, 14 and 30 days) using the Baermann funnel method. Then, RNA was extracted and cDNA of *PWN* was synthesized (see Section 4.4). Quantitative reverse transcription PCR (qRT-PCR) was performed to determine the expression level of *BxATG1* and *BxATG8*.

### 4.9. BxATG1 and BxATG8 Interference Using Double-Stranded RNA

Double-stranded RNA (dsRNA) was synthesized using the MEG script RNAi Kit (Ambion Inc., Austin, TX, USA) with the primers *BxATG1-T7I-F*, *BxATG1-I-R*, *BxATG1-I-F*, *BxATG1-T7I-R*, *BxATG8-T7I-F*, *BxATG8-I-R*, *BxATG8-I-F*, *BxATG8-T7I-R*, *GFP-T7I-F*, *GFP-I-R*, *GFP-I-F*, and *GFP-T7I-R* (Table 2). The RNAi soaking method was performed according to Urwin et al. [53]. Freshly cultured nematodes were soaked in dsRNA solution (800 ng/µL) and incubated at 180 rpm for 48 h at 20 °C. The nematodes soaked in the corresponding non-dsRNA solution were used as controls. Each treatment had three replicates. Samples from each treatment were washed thoroughly with ddH_2_O three times after soaking and then used for additional experiments. *BxATG1* and *BxATG8* were silenced by RNAi effectively which was performed according to Deng et al. [21].

### 4.10. Evaluation of Virulence of PWN after RNAi

To determine the virulence of *PWN* after RNAi (*BxATG1* and *BxATG8*), the nematodes were inoculated onto *P. thunbergii* seedlings for virulence determination. One thousand nematodes were injected into each seedling. *PWN* soaked in ds*GFP* and ddH_2_O were used as negative controls, ddH_2_O alone as a positive control. Eight biological replicates were conducted. Periodically, the seedlings were observed and were photographed to record their state. The disease severity of *P. thunbergii* seedlings was divided into five levels: 0, healthy seedlings with green needles growing well; I, a few needles turning brown; II, half of the needles turning brown and the terminal shoots of seedlings bending; III, most of the needles turning brown, and the terminal shoots of seedlings drooping; and IV, all of the needles turning brown, and the whole seedling wilting. The infection rates and the disease severity index were calculated according to Xiang et al. [47].

$$\mathrm{Infection}\;\mathrm{rates}=\frac{\sum \;\mathrm{number}\;\mathrm{of}\;\mathrm{infected}\;\mathrm{plants}\;\mathrm{with}\;\mathrm{symptoms}\;}{\mathrm{Total}\;\mathrm{number}\;\mathrm{of}\;\mathrm{plants}}\times 100\%$$

$$\mathrm{Disease}\;\mathrm{severity}\;\mathrm{index}\;(\mathrm{DSI})\;=\;\frac{\sum \;\mathrm{number}\;\mathrm{of}\;\mathrm{disease}\;\mathrm{plants}\times \;\mathrm{symptom}\;\mathrm{stage}}{\mathrm{Total}\;\mathrm{number}\;\mathrm{of}\;\mathrm{plants}\times \;\mathrm{highest}\;\mathrm{symptom}\;\mathrm{stage}}\times \;100$$

### 4.11. Statistical Analysis

All assays were performed in triplication. The statistical significance was determined using SPSS Statistics 17.0 software (IBM China Company Ltd., Beijing, China) to perform the paired *t*-tests. Asterisks indicate statistically significant differences (** *p* < 0.01, Student’s *t*-test).

## Figures and Tables

**Table 1:** The symptoms of *P. thunbergii* inoculated with *PWN*.

Nematodes	Infection Rates			Disease Severity Indices			Days of Symptoms Appeared (d)	Days of *P. Thunbergii* Wilted (d)
	14th day	20th day	30th day	14th day	20th day	30th day		
nematodes soaked in ddH_2_O	50	100	100	12.5	50	100	12	28
nematodes soaked in ds*GFP*	50	100	100	12.5	50	100	12	29
nematodes soaked in ds*BxATG1*	0	50	100	0	12.5	56.3	17	45
nematodes soaked in ds*BxATG8*	0	75	100	0	18.8	87.5	16	38

**Table 2:** PCR primers used in the study.

Name of Primers	Sequence (5′–3′)
*BxATG1-T7I-F*	TAATACGACTCACTATAGGGAAGGCAGAAATCGGACA
*BxATG1-I-R*	AATCGGCTCATGGAAAA
*BxATG1-I-F*	AAGGCAGAAATCGGACA
*BxATG1-T7I-R*	TAATACGACTCACTATAGGGAATCGGCTCATGGAAAA
*BxATG8-T7I-F*	TAATACGACTCACTATAGGGAACCCAAGTTTGAGACCT
*BxATG8-I-R*	TAATACGACTCACTATAGGGAAAGGAGAAGAACTTTTCAC
*BxATG8-I-F*	CTGTTACAAACTCAAGAAGG
*BxATG8-T7I-R*	AAAGGAGAAGAACTTTTCAC
*GFP-T7I-F*	TAATACGACTCACTATAGGGCTGTTACAAACTCAAGAAGG
*GFP-I-R*	CGAAAACACTACAATAAGA
*GFP-I-F*	AACCCAAGTTTGAGACCT
*GFP-T7I-R*	TAATACGACTCACTATAGGG CGAAAACACTACAATAAGA
*Actin F*	GCAACACGGAGTTCGTTGTAGA
*Actin R*	GTATCGTCACCAACTGGGATGA
*qBxATG1-F*	AGAGTGTTGGGTGAGGGA
*qBxATG1-R*	CTCGGCATTGGTACATTATA
*qBxATG8-F*	GTCAACGATGTCATTCCCCA
*qBxATG8-R*	AACTGATCACTCTTCGGCGG

## References

[1] Mamiya Y. (1988). History of pine wilt disease in Japan. J. Nematol..

[2] Mota M.M., Futai K., Vieira P. (2009). Pine wilt disease and the pinewood nematode, *Bursaphelenchus xylophilus*. Integrated Management and Biocontrol of Vegetable and Grain Crops Nematodes.

[3] Robertson L., Arcos S.C., Escuer M., Merino R.S., Esparrago G., Abelleira A., Navas A. (2011). Incidence of the pine wood nematode *Bursaphelenchus xylophilus* Steiner & Buhrer, 1934 (Nickle, 1970) in Spain. Nematology.

[4] Yang B.J., Pan H.Y., Tang J., Wang Y.Y., Wang L.F. (2003). Pine Wood Nematode Disease.

[5] Zhang K., Liang J.Y., Dong H., Zhang X.Y. (2010). Research advances of pine wood nematode disease in China. World For. Res..

[6] Wang S.L., Cao F.X., Wang M., Qian W.Q. (2009). Research Advance of *Bursaphelenchus xylophilus* Genes. J. Cent. South Univ. For. Technol..

[7] Reggiori F., Klionsky D.J. (2002). Autophagy in the eukaryotic cell. Eukaryot. Cell.

[8] Zhang L. (2007). Cloning and Functional Analysis of *MgATG3*, *MgATG4* and *MgATG7* Gene in *Magnaporthe grisea*. Master’s Thesis.

[9] Liu X., Lu J., Lin F. (2007). Autophagy during conidiation, conidial germination and turgor generation in *Magnaporthe grisea*. Autophagy.

[10] Dong B., Liu X.H., Lu J.P. (2009). MgAtg9 trafficking in *Magnaporthe oryzae*. Autophagy.

[11] Gao H.M., Liu X.G., Shi H.B. (2013). MoMon1 is required for vacuolar assembly, conidiogenesis and pathogenicity in the rice blast fungus *Magnaporthe oryzae*. Res. Microbiol..

[12] Klionsky D.J., Cregg J.M., Dunn W.A., Emr S.D., Sakai Y., Sandoval I.V., Sibirny A., Subramani S., Thumm M., Veenhuis M. (2003). A unified nomenelature for yeast autophagy-related genes. Dev. Cell.

[13] Meléndez A., TaUóczy Z., Seaman M., Eskelinen E.L., Hall D.H., Levine B. (2003). Autophagy genes are essential for dauer development and life-span extension in *C. elegans*. Science.

[14] Guo B. (2014). Genome-Wide Screen to Identify Regulators of Autophagy Activity in *C. elegans*. Ph.D. Thesis.

[15] Gao H., Li Y.Y., Huang R., Wu S.Y. (2015). Application progress of model organisms *C. elegans* in the study of autophagy-related diseases. J. Parasit. Biol..

[16] Zhang H. (2014). The Function of *C. elegans* ATG-16 Homolog in the Autophagy Pathway. Ph.D. Thesis.

[17] Sarkar S. (2013). Regulation of autophagy by mTOR-dependent and mTOR-independent pathways: Autophagy dysfunction in neurodegenerative diseases and therapeutic application of autophagy enhancers. Biochem. Soc. Trans..

[18] Shpilka T., Weidberg H., Pietrokovski S., Elazar Z. (2011). Atg8: An autophagy-related ubiquitin-like protein family. Genome Biol..

[19] Loewith R., Jacinto E., Wullschleger S., Lorberg A., Crespo J.L., Bonenfant D., Oppliger W., Jenoe P., Hall M.N. (2002). Two TOR complexes, only one of which is rapamycin sensitive, have distinct roles in cell growth control. Mol. Cell.

[20] Caccamo A., Majumder S., Richardson A., Strong R., Oddo S. (2010). Molecular interplay between mammalian target of rapamycin (mTOR), amyloid-β, and tau effects on cognitive impairments. J. Biol. Chem..

[21] Deng L.N., Wu X.Q., Ye J.R., Xue Q. (2016). Identification of autophagy in the pine wood nematode *Bursaphelenchus xylophilus* and the molecular characterization and functional analysis of two novel autophagy-related genes, *BxATG1* and *BxATG8*. Int. J. Mol. Sci..

[22] Liu X.H., Lu J.P., Zhang L., Dong B., Min H., Lin F.C. (2007). Involvement of a *Magnaporthe grisea* serine/threonine kinase gene, *MgATG1*, in appressorium turgor and pathogenesis. Eukaryot. Cell.

[23] Bryant B., Raikhel A.S. (2011). Programmed autophagy in the fat body of Aedesaegypti is required to maintain egg maturation cycles. PLoS ONE.

[24] Asakura M., Ninomiya S., Sugimoto M., Oku M., Yamashita S.I., Okuno T., Sakai Y., Takano Y. (2009). Atg26-mediated pexophagy is required for host invasion by the plant pathogenic fungus *Colletotrichum orbiculare*. Plant Cell.

[25] Boya R., Reggiori R., Codogno P. (2013). Emerging regulation and functions of autophagy. Nat. Cell Biol..

[26] Ma K.X., Xi X.Z. (2011). The mechanism and function of autophagy. Biol. Teach..

[27] Li M. (2009). Regulation of mTOR Signaling by Reactive Oxygen Species and Its Mechanisms. Ph.D. Thesis.

[28] Scherz-Shouval R., Shvets E., Fass E., Shorer H., Gil L., Elazar Z. (2007). Reactive oxygen species are essential for autophagy and specifically regulate the activity of Atg4. EMBO J..

[29] Liu J., Wu X.Q., Ying C.X., He L.X., Ye J.R. (2009). The difference of progenitive power and superoxide anion production in *Bursaphelenchus xylophilus* and *B. mucronatus*. J. Nanjing For. Univ..

[30] Yu L.Z., Wu X.Q., Ye J.R., Zhang S.N. (2013). Relationships between nitric oxide response signal and external factors during the early interaction between *Pinus thunbergii* and *Bursaphelenchus xylophilus*. Chin. J. Appl. Ecol..

[31] Yu L.Z., Wang C., Lu M. (2015). The Role of ROS signaling molecules in plant disease resistance response. Shanxi For. Sci. Technol..

[32] Yang Z., Klionsky D.J. (2009). An overview of the molecular mechanism of autophagy. Curr. Top. Microbiol. Immunol..

[33] Khalfan W.A., Klionsky D.J. (2002). Molecular machinery required for autophagy and the cytoplasm to vacuole targeting (Cvt) pathway in *S. cerevisiae*. Curr. Opin. Cell Biol..

[34] Suzuki K. (2013). Selective autophagy in budding yeast. Cell Death Differ..

[35] Mizushima N. (2007). Autophagy: Process and function. Genes Dev..

[36] Espada M., Silva A.C., van den Akker S.E., Cock P.J., Mota M., Jones J.T. (2016). Identification and characterization of parasitism genes from the pinewood nematode *Bursaphelenchus xylophilus* reveals a multilayered detoxification strategy. Mol. Plant Pathol..

[37] Kariola T., Brader G., Li J., Palva E.T. (2005). Chlorophyllase L, a damage control enzyme, affects the balance between defense pathways in plants. Plant Cell.

[38] Yu L.Z., Wu X.Q., Ye J.R., Zhang S.N., Wang C. (2012). NOS-like-mediated nitric oxide is involved in *Pinus thunbergii* response to the invasion of *Bursaphelenchus xylophilus*. Plant Cell Rep..

[39] Shinya R., Morisaka H., Takeuchi Y., Ueda M., Futai K. (2010). Comparison of the surface coat proteins of the pine wood nematode appeared during host pine infection and in vitro culture by a proteomic approach. Phytopathology.

[40] Klionsky D.J. (2005). The molecular machinery of autophagy: Unanswered questions. J. Cell Sci..

[41] Wesley S.V., Helliwell C.A., Smith N.A., Wang M.B., Rouse D.T., Liu Q., Gooding P.S., Singh S.P., Abbott D., Stoutjesdijk P.A. (2001). Construct design for efficient, effective and high-throughput gene silencing in plants. Plant J..

[42] Chang C.H., Wang H.I., Lu H.C., Chen C.E., Chen H.H., Yeh H.H., Tang C.Y. (2012). An efficient RNA interference screening strategy for gene functional analysis. BMC Genom..

[43] Ma H.B., Lu Q., Liang J., Zhang X.Y. (2011). Functional analysis of the cellulose gene of the pine wood nematode, *Bursaphelenchus xylophilus*, using RNA interference. Genet. Mol. Res..

[44] Kang J.S., Koh Y.H., Moon Y.S., Lee S.H. (2012). Molecular properties of a venom allergen-like protein suggest a parasitic function in the pinewood nematode *Bursaphelenchus xylophilus*. Int. J Parasitol..

[45] Cheng X.Y., Dai S.M., Xiao L., Xie B.Y. (2010). Influence of cellulase gene knockdown by dsRNA interference on the development and reproduction of the pine wood nematode *Bursaphelenchus xylophilus*. Nematology.

[46] Xu X.L., Wu X.Q., Ye J.R., Huang L. (2015). Molecular characterization and functional analysis of three pathogenesis-related Cytochrome *P450* genes from *Bursaphelenchus xylophilus* (Tylenchida: Aphelenchoidoidea). Int. J. Mol. Sci..

[47] Xiang Y., Wu X.Q., Zhou A.D. (2015). Bacterial diversity and community structure in the pine wood nematode *Bursaphelenchus xylophilus* and *B. mucronatus* with different virulence by high-throughput sequencing of the 16S rDNA. PLoS ONE.

[48] Viglierchio D.R., Schmitt R.V. (1983). On the methodology of nematode extraction from field samples: Baermann funnel modifications. J. Nematol..

[49] Jiang L.L. (2014). The Effects of Rapamycin on Autophagy and Renal Interstitial Fibrosis of UUO Rats. Master’s Thesis.

[50] Li J.B., Deng Y.J., Zhang X.P. (2009). Influence of rapamycin on autophagy and apoptosis of two lepidopteran cell lines. Chin. Bull. Entomol..

[51] Livak K., Schmittgen D. (2001). Analysis of relative gene expression data using real-time quantitative PCR and the 2^–ΔΔ*C*t^ method. Methods.

[52] Zhu L.H., Ye J.R., Negi S., Xu X.L., Wang Z.L., Ji J.Y. (2012). Pathogenicity of aseptic *Bursaphelenchus xylophilus*. PLoS ONE.

[53] Urwin P.E., Lilley C.J., Atkinson H.J. (2002). Ingestion of double-stranded RNA by preparasitic juvenile cystnematodes leads to RNA interference. Mol. Plant Microbe Interact..

